# Preterm prelabour rupture of membranes before 23 weeks’ gestation: prospective observational study

**DOI:** 10.1136/bmjmed-2023-000729

**Published:** 2024-03-19

**Authors:** Laura Goodfellow, Angharad Care, Ciara Curran, Devender Roberts, Mark A Turner, Marian Knight, Alfirevic Zarko

**Affiliations:** 1 Women's and Children's Health, University of Liverpool, Liverpool, UK; 2 Little Heartbeats Patient Support Group, Buxton, UK; 3 Liverpool Women's Hospital NHS Foundation Trust, Liverpool, UK; 4 National Perinatal Epidemiology Unit, Oxford, UK

**Keywords:** Prenatal care, Pregnancy complications, Neonatology

## Abstract

**Objective:**

To describe perinatal and maternal outcomes of preterm prelabour rupture of membranes (PPROM) before 23 weeks' gestation in a national cohort.

**Design:**

Prospective observational study.

**Setting:**

National population based cohort study with the UK Obstetric Surveillance System (UKOSS), a research infrastructure of all 194 obstetric units in the UK, 1 September 2019 to 28 February 2021.

**Participants:**

326 women with singleton and 38 with multiple pregnancies with PPROM between 16+0 and 22+6 weeks+days' gestation.

**Main outcome measures:**

Perinatal outcomes of live birth, survival to discharge from hospital, and severe morbidity, defined as intraventricular haemorrhage grade 3 or 4, or requiring supplemental oxygen at 36 weeks' postmenstrual age, or both. Maternal outcomes were surgery for removal of the placenta, sepsis, admission to an intensive treatment unit, and death. Clinical data included rates of termination of pregnancy for medical reasons.

**Results:**

Perinatal outcomes were calculated with all terminations of pregnancy for medical reasons excluded, and a worst-best range was calculated assuming that all terminations for medical reasons and those with missing data would have died (minimum value) or all would be liveborn (maximum value). For singleton pregnancies, the live birth rate was 44% (98/223), range 30-62% (98/326-201/326), perinatal survival to discharge from hospital was 26% (54/207), range 17-53% (54/326-173/326), and 18% (38/207), range 12-48% (38/326-157/326) of babies survived without severe morbidity. The rate of maternal sepsis was 12% (39/326) in singleton and 29% (11/38) in multiple pregnancies (P=0.004). Surgery for removal of the placenta was needed in 20% (65/326) and 16% (6/38) of singleton and twin pregnancies, respectively. Five women became severely unwell with sepsis; two died and another three required care in the intensive treatment unit.

**Conclusions:**

In this study, 26% of women who had very early PPROM with expectant management had babies that survived to discharge from hospital. Morbidity and mortality rates were high for both mothers and neonates. Maternal sepsis is a considerable risk that needs more research. These data should be used in counselling families with PPROM before 23 weeks' gestation, and currently available guidelines should be updated accordingly.

**WHAT IS ALREADY KNOWN ON THIS TOPIC**

- Preterm prelabour rupture of membranes (PPROM) before 23 weeks' gestation is a serious complication of pregnancy with high rates of morbidity for mothers and babies
- Women with PPROM before 23 weeks’ gestation are often advised to consider termination of the pregnancy for medical reasons
- Recent population based pregnancy outcomes are not available, making counselling difficult

**WHAT THIS STUDY ADDS**

- This study identified substantial maternal morbidity; 14% of women developed sepsis and two (0.5%, 95% confidence interval 0.15% to 2.0%) died
- Immediate termination of pregnancy did not always mitigate the risk of maternal sepsis
- A substantial minority of babies survived; 26% of expectantly managed babies survived to discharge from hospital and the potential worst-best case survival range, including terminations for medical reasons, was 17-53%
- The live birth rate improved with later gestational age at PPROM
- A trend towards improved survival of babies to hospital discharge without severe morbidity with increasing gestational age at PPROM was seen, but this was more strongly correlated with gestational age at birth

**HOW THIS STUDY MIGHT AFFECT RESEARCH, PRACTICE OR POLICY**

- This population based study has described the outcomes of pregnancies affected by PPROM before 23 weeks' gestation
- These data should be used as a baseline to support research into the complex pathologies, including sepsis, of pregnancies affected by early PPROM
- Understanding these results is imperative to provide appropriate counselling and management of this difficult complication

## Introduction

Preterm prelabour rupture of membranes (PPROM) complicates 30-40% of all preterm births.^1^ Serious complications of PPROM include chorioamnionitis, leading to maternal or neonatal sepsis, or both, placental abruption, and stillbirth. UK clinical guidelines describe the management of this condition but only for pregnancies after 24 weeks' gestation (ie, when pregnancy is legally viable).^1^ Before this gestational age, the burden of neonatal morbidity and mortality has been considered so high that termination of pregnancy is generally offered because the possibility of survival of the fetus is low and also because of concerns about lifelong neurological disability as a result of extreme prematurity.^2^ The incidence of PPROM before 23 weeks' gestation is low (~0.1%), and therefore a typical obstetric unit with 4000 births a year will manage fewer than five incidences annually,^3^ resulting in a paucity of both research and clinical experience in expectant management of this condition.

**Figure 1:**
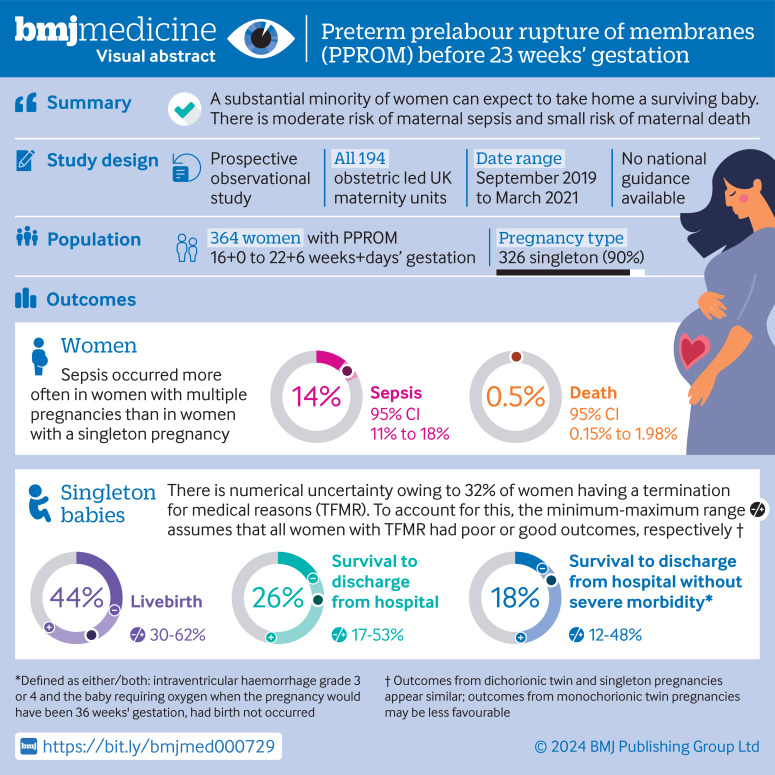
Visual abstract

Families report that clinical care in the UK, including offering termination of pregnancy for medical reasons, differs broadly in seemingly similar clinical scenarios.^4^ These inconsistencies understandably add to parental distress under already difficult circumstances. The aim of this study was to provide UK population level data for pregnancies with PPROM between 16+0 and 22+6 weeks+days' gestation, grouped according to gestational age when PPROM occurred. The study was carried out with the UK Obstetric Surveillance System (UKOSS), a research infrastructure that includes every consultant led maternity department in the UK. Figure 1 shows the visual abstract.

## Method

### Data collection

The study included women with PPROM between 16+0 and 22+6 weeks’ gestation from 1 September 2019 to 28 February 2021 (inclusive). Data collection was extended by six months from the original planned period of one year, when the covid-19 pandemic was declared in the UK in March 2020, to investigate potential changes in outcomes secondary to the pandemic. This paper uses the term "woman" and includes all female people, including those who do not see themselves as women.

UKOSS is a research platform that collects population based information about rare pregnancy events from all 194 consultant led maternity hospitals in the UK.^5^ Because of the comprehensive coverage of NHS maternity care, a reasonable assumption is that pregnancies with very early PPROM in the UK would have been treated at one or more of these hospitals. Nominated reporting clinicians^6^ notified UKOSS of all pregnancies with PPROM between 16+0 and 22+6 weeks' gestation (inclusive). Two exclusion criteria were pregnancies where membranes ruptured before 16+0 weeks' gestation but were only diagnosed in the 16+0-22+6 week period, and pregnancies where intrauterine death of all fetuses was diagnosed before rupture of membranes.

To capture all relevant pregnancies, the minimum time between PPROM and labour or birth was not specified. After UKOSS received a report of an eligible pregnancy, information was requested with a set proforma for data collection,^7^ with regular reminders to return missing data at six, 10, 14, and 28 weeks after notification. A final reminder was sent at the end of the data collection period in September 2021. If a woman was still pregnant when the initial data collection form was received, pregnancy outcome data were requested at two, six, 10, 14, and 28 weeks after the estimated due date. Referring hospitals that the woman or baby were transferred to were also contacted to request outcome data.

### Sample size and statistical analysis

Our study was a time limited national observational study and therefore no formal power calculation was carried out. The incidence rate was calculated based on the denominator of maternities (ie, the number of pregnancies resulting in the birth of one or more liveborn or stillborn babies) in 2020 from the constituent nations of the UK.^8–10^ Statistical analysis was performed in Stata version 15.1 (StataCorp).^11^ The study is reported in accordance with the Strengthening the Reporting of Observational Studies in Epidemiology (STROBE) statement: guidelines for reporting observational studies.^12^

Personal characteristics are reported for the whole cohort and according to whether the pregnancy had expectant management or was terminated for medical reasons. Pregnancies that were terminated for medical reasons after a period of expectant management were included in the termination of pregnancy for medical reasons group. Gestational age at PPROM and at birth were calculated from ultrasound assessment of estimated date of delivery.

Maternal age at estimated date of delivery was calculated assuming a date of birth of 1 June within the given year of birth because only maternal year of birth was collected to maintain anonymity. Body mass index was based on first recorded weight and height in pregnancy. Ethnic group was recorded from medical records, based on self-reports. An adverse pregnancy history was a previous pregnancy with PPROM between 16+0 and 33+6 weeks' gestation, mid-trimester loss between 16+0 and 22+6 weeks' gestation, or spontaneous preterm birth between 23+0 and 36+6 weeks' gestation.

The effect of the covid-19 pandemic on pregnancy outcomes was assessed by grouping singleton pregnancies into those with PPROM between 1 September 2019 and 29 February 2020 (before covid-19) and those between 1 March 2020 and 28 February 2021 (during the covid-19 pandemic). The rate of reported pregnancies and perinatal and maternal pregnancy outcomes were compared across the two groups.

Pregnancy outcomes for babies and mothers are reported separately for singleton and twin pregnancies. Higher order multiples are briefly described. Singleton pregnancy outcomes were divided into four mutually exclusive groups based on when PPROM occurred: 16+0-17+6, 18+0-19+6, 20+0-21+6, and 22+0-22+6 weeks’ gestation. The 22+0-22+6 weeks’ gestation group was analysed separately because guidelines from the British Association of Perinatal Medicine, produced in October 2019, suggest that in some pregnancies, with parental agreement, active resuscitation should be considered at birth from 22+0 weeks' gestation.^13^ Twin pregnancy outcomes are presented according to chorionicity of the pregnancy.

The time between PPROM and birth was calculated for all, except those who had a termination of pregnancy for medical reasons, and included spontaneous births, intrauterine deaths, and medically indicated births. Maternal outcomes were sepsis, surgery for removal of the placenta, admission to the intensive treatment unit, and death. All maternal outcomes are reported as defined by local clinicians. Perinatal outcomes included live births, survival to hospital discharge, and survival to hospital discharge without severe morbidity. Severe morbidity was defined according to Kibel et al as intraventricular haemorrhage grade 3 or 4, or requiring supplemental oxygen at 36 weeks' postmenstrual age, or both.^14^ Also, reporting clinicians were asked to record whether the babies had limb contractures, neonatal seizures, or severe lung disease during the neonatal period. Severe lung disease was defined as requiring high frequency oscillatory ventilation or inhaled nitric oxide during admission to hospital in the neonatal period, or supplemental oxygen at 36 weeks' postmenstrual age.

To account for the effect of termination of pregnancy for medical reasons and missing data on the calculated rates of perinatal outcomes, three rates were calculated: perinatal outcome in expectantly managed pregnancies with known outcome for the baby; perinatal outcome assuming that all babies of pregnancies that had termination of pregnancy for medical reasons or an unknown outcome would have died; and perinatal outcome assuming all babies of pregnancies that had termination of pregnancy for medical reasons or an unknown outcome would have survived. Details of pregnancy loss are described based on five mutually exclusive groups: birth or intrauterine death before 22+0 weeks' gestation (often called miscarriage); stillbirth at or after 22+0 weeks' gestation; neonatal death; termination of pregnancy for medical reasons without expectant management; and termination of pregnancy for medical reasons after a period of expectant management.

Data are presented as descriptive statistics (mean (standard deviation) and median (interquartile range)) and differences between groups were compared with the t test for maternal age, Mann-Whitney U test for gestational age at PPROM, and χ^2^ tests for categorical variables. Maternal death and admission to the intensive treatment unit according to covid-19 pandemic status were compared with Fisher's exact tests. The Wilson score interval was used to generate confidence intervals for proportions where appropriate. A P value <0.05 was considered significant.

### Patient and public involvement

The patient support and advocacy group, Little Heartbeats, approached one of the authors (AC) with concerns about inconsistency in counselling, management, and maternal outcomes of instances of PPROM before 23 weeks' gestation, stimulating this research. Author CC (the founder of Little Heartbeats) and the patient and public members of the UKOSS Steering Committee were then involved in the design of the study, conduct of the study, and interpretation of the results. CC met regularly with authors LG and AC to review the findings and plan the optimal presentation of the data. The completed analysis was also reviewed by patient and public representation within the UKOSS steering committee.

The women who experienced PPROM were reported anonymously by nominated hospital reporting clinicians to the study team, as per the study’s ethical approval. It is therefore not possible for the study team to communicate the results of this work directly to the women involved. The study team have produced two infographics describing the findings; one for women and families and one for healthcare professionals. These will be available on the Univeristy of Liverpool website and shared via Little Heartbeats social media, the UKOSS newsletter and the Wellbeing of Women social media. We are also planning an information sharing day in Liverpool for patients in 2024.

## Results

All 194 UK hospitals participated in UKOSS, and 125 (64%) hospitals reported at least one pregnancy with PPROM at 16+0-22+6 weeks’ gestation, resulting in 551 women in the study. Of these 551 women, 54 were subsequently identified by reporting clinicians as not meeting the case definition (n=50) or were a duplicate report (n=4). A further six women were removed because of missing notes and in 75 instances the data collection form was not completed. The analysis team then excluded a further 48 women from the analysis for ineligibility, duplication, or insufficient information to assess eligibility (online supplemental figure A1).

10.1136/bmjmed-2023-000729.supp1 Supplementary data

In total, 368 women met the inclusion criteria, of whom 38 had multiple pregnancies (online supplemental figure A1). An estimated 1 011 924 maternities were reported in the UK for a period of 18 months^8–10^ and hence the estimated incidence of PPROM at 16+0-22+6 weeks’ gestation was 1 in 2750 maternities (0.04%). In 364 pregnancies, information was available for neonatal and maternal pregnancy outcomes and this group formed the full analysis cohort (online supplemental figure A1).

### Personal characteristics

Maternal characteristics were similar in the expectant management group and the termination of pregnancy for medical reasons group. An earlier median gestational age of PPROM was reported in the termination for medical reasons group (table 1).

**Table 1:** Personal characteristics of cohort, presented for whole cohort and according to expectant management or termination of pregnancy for medical reasons

Characteristics	Whole cohort (n=364)	Management group		Comparison of management groups (P value)
		Expectant (n=251)	Termination for medical reasons (n=113)	
Mean (SD) maternal age (years)	32 (6)	32 (6)	33 (5)	0.265
Maternal smoking at booking appointment	81 (22)	61 (24)	20 (18)	0.151
Maternal body mass index:				
<18.5	11 (3)	9 (4)	2 (2)	0.805
18.5-<25.0	134 (37)	95 (38)	39 (35)	
25.0-<30.0	92 (25)	59 (24)	33 (29)	
30.0-<35	56 (15)	39 (16)	17 (15)	
≥35.0	51 (14)	36 (14)	15 (13)	
Not specified	20 (5)	13 (5)	7 (6)	
Maternal ethnic group:				
Asian	62 (17)	42 (17)	20 (18)	0.644
Black	35 (10)	23 (9)	12 (11)	
Mixed or any other ethnic group	14 (4)	10 (4)	4 (4)	
White	248 (68)	171 (68)	77 (68)	
Not specified	5 (1)	5 (2)	0 (0)	
Obstetric history:				
Primiparous	155 (43)	104 (41)	51 (45)	0.569
At least one previous pregnancy affected by PPROM, mid-trimester loss, or preterm birth	63 (17)	40 (16)	23 (20)	0.303
Singleton pregnancy	326 (90)	223 (89)	103 (91)	0.506
Amniocentesis or chorionic villous sampling in pregnancy	10 (3)	6 (2)	4 (4)	0.535
Cervical cerclage before PPROM	29 (8)	22 (9)	7 (6)	0.402
Median (IQR) gestational age at PPROM (weeks+days)	19+3 (17+6-21+1)	19+6 (18+2-21+2)	18+6 (17+2-20+2)	0.000

Data are number (%) unless indicated otherwise. IQR=interquartile range; PPROM=preterm prelabour rupture of membranes; SD=standard deviation.

### Covid-19 pandemic

The study included 137 women with PPROM in singleton pregnancies in the six months before the covid-19 pandemic and 189 in the year during the pandemic. The incidence of reported PPROM was higher before the pandemic than during the pandemic (median 23, interquartile range 19.5-24.5 incidences/month *v* 16.5, 14-20.25, respectively; P<0.001). The number of reported incidences each month was lowest for the period July 2020-December 2020 (online supplemental figure A2). We found no significant differences in maternal or perinatal outcomes according to whether PPROM occurred before or during the pandemic (online supplemental tables A1 and A2), and hence the remainder of the analysis was based on the whole dataset.

### Termination of pregnancy for medical reasons

Termination of pregnancy for medical reasons was performed for 32% of singleton pregnancies (103/326) and 32% of multiple pregnancies (12/38). Among singleton pregnancies, 62 terminations were performed without a period of expectant management and 41 after initial expectant management (table 2). Among multiple pregnancies, 10 terminations for medical reasons were performed after a period of expectant management after PPROM, including six (6/38, 16%) where death of a single baby (either intrauterine death or spontaneous birth before 22+0 weeks' gestation) was followed by termination of pregnancy for medical reasons of a second twin.

**Table 2:** Perinatal outcomes of singleton pregnancies, focusing on babies that died

Perinatal outcomes	Whole cohort (n=326)*	Gestational age at PPROM (weeks+days)				Unadjusted relative rate ratio of outcome for each additional week of gestation at PPROM (RR (95% CI), P value)
		16+0-17+6 (n=82)	18+0-19+6 (n=102)	20+0-21+6 (n=105)	22+0-22+6 (n=37)	
Termination for medical reasons without expectant management	62 (19)	25 (30)	19 (19)	17 (16)	1 (3)	0.75 (0.64 to 0.87), 0.001
Termination for medical reasons after expectant management	41 (13)	14 (17)	13 (13)	8 (8)	6 (16)	0.92 (0.77 to 1.09), 0.33
Birth or intrauterine death <22+0 weeks' gestation (miscarriage) (expectant management throughout pregnancy)	90/223 (40)	26/33 (79)	37/70 (53)	27/80 (34)	—	0.80 (0.70 to 0.91), 0.001
Intrauterine deaths ≥22+0 weeks' gestation (expectant management throughout pregnancy)	35/223 (16)	3/33 (9)	6/70 (9)	16/80 (20)	10/30 (33)	1.59 (1.27 to 1.99), 0.001
Neonatal deaths (expectant management throughout pregnancy)	28/223 (13)	4/33 (12)	8/70 (11)	10/80 (13)	6/30 (20)	1.24 (1.00 to 1.53), 0.05

Data are No (%) or No/total No (%). CI=confidence interval; PPROM=preterm prelabour rupture of membranes; RR=relative rate.

*Gestational age at PPROM 16+0-22+6 weeks+days.

Among the 62 singleton pregnancies that had termination of pregnancy for medical reasons without a period of expectant management, median time from PPROM to termination was two days (interquartile range 1-3.5). The indication for termination of pregnancy for medical reasons without a period of expectant management was on the advice of a clinician because of severe chorioamnionitis or sepsis in 24% (15/62) of pregnancies, patient choice based on poor maternal or neonatal outcomes in 73% (45/62), and two were performed for other reasons.

Median time between PPROM and termination of pregnancy for medical reasons for the 41 singleton pregnancies that had a period of expectant management was five days (interquartile range 3-13). In 34% (14/41) of these pregnancies, the decision was based on the advice of a clinician because of severe chorioamnionitis or sepsis, and the remaining 66% (27/41) of terminations were a result of patient choice based on the likelihood of poor maternal or neonatal outcomes.

The rate of termination of pregnancy for medical reasons without expectant management reduced from 30% (25/82) when PPROM occurred at 16+0-17+6 weeks’ gestation to 3% (1/37) with PPROM at 22+0-22+6 weeks’ gestation. We found a reduction of 0.75 in the relative rate ratio for the likelihood of termination of pregnancy for medical reasons without expectant management for each additional week of gestational age at PPROM (95% confidence interval 0.64 to 0.87, P=0.001). The rate of termination of pregnancy for medical reasons after expectant management was relatively similar across gestational ages (8-17%) (table 2).

### Pregnancy after PPROM

We calculated the time between PPROM and birth in 220/223 (99%) expectantly managed singleton pregnancies. Median time between PPROM and birth was 13 days (interquartile range 3-50). Median gestational age at birth was 23+0 weeks (interquartile range 20+2-27+4) (table 3).

**Table 3:** Perinatal outcomes and gestational age at birth of singleton pregnancies, focusing on babies that survived

Perinatal outcomes	Whole cohort (n=326)*	Gestational age at PPROM (weeks+days)				Unadjusted relative rate ratio of outcome for each additional week of gestation at PPROM (RR (95% CI), P value)
		16+0-17+6 (n=82)	18+0-19+6 (n=102)	20+0-21+6 (n=105)	22+0-22+6 (n=37)	
Median (IQR) gestational age at birth (weeks+days)	23+0 (20+2-27+4)	19+2 (17+2-27+6)	21+3 (19+4-27+4)	23+1 (21+2-26+6)	24+2 (22+6-28+1)	—
Live births:						
No/total No (%) expectant management‡	98/223 (44)	14/43 (33)	27/70 (39)	37/80 (46)	20/30 (67)	1.25 (1.08 to 1.45), 0.004
Worst-best possible outcome (range) (minimum No/total No (%) to maximum No/total No (%)) §	98/326 (30) to 201/326 (62)	14/82 (17) to 53/82 (65)	27/102 (26) to 59/102 (58)	37/105 (35) to 62/105 (59)	20/37 (54) to 27/37 (73)	
Median (IQR) gestational age at birth (weeks+days)	28+2 (25+2-30+2)	28+4 (27+6-34+2)	28+4 (26+4-30+1)	27+1 (23+4-30+4)	26+3 (23+1-30+1)	
Survival to hospital discharge:						
No/total No (%) total expectant management†‡	54/207 (26)	7/40 (18)	16/67 (24)	21/74 (28)	10/26 (38)	1.19 (1.00 to 1.42), 0.06
Worst-best possible outcome (range) (minimum No/total No (%) to maximum No/total No (%)) §	54/326 (17) to 173/326 (53)	7/82 (9) to 49/82 (60)	16/102 (16) to 51/102 (50)	21/105 (20) to 52/105 (50)	10/37 (27) to 21/37 (57)	
Median (IQR) gestational age at birth (weeks+days)	29+4 (27+1-34+2)	34+2 (29+0-36+4)	28+5 (27+2-33+6)	30+2 (26+3-33+5)	29+2 (27+6-31+1)	
Survival to hospital discharge without severe morbidity:						
No/total No (%) expectant management†‡	38/207 (18)	5/40 (13)	11/67 (16)	13/74 (18)	9/26 (35)	1.21 (0.99 to 1.48), 0.07
Worst-best possible outcome (range) (minimum No/total No (%) to maximum No/total No (%)) §	38/326 (12) to 157/326 (48)	5/82 (6) to 51/82 (62)	11/102 (11) to 46/102 (45)	13/105 (12) to 44/105 (42)	9/37 (24) to 20/37 (54)	
Median (IQR) gestational age at birth (weeks+days)	30+2 (28+2-35+5)	34+3 (34+2-36+4)	28+5 (26+0-35+5)	31+5 (28+4–35+5)	30+1 (28+2-31+1)	

CI=confidence interval; IQR=interquartile range; PPROM=preterm prelabour rupture of membranes; RR=relative rate.

*Gestational age at PPROM 16+0-22+6 weeks+days.

†Excludes pregnancies with babies that were live born at ≥22+0 weeks' gestational age with missing discharge status. PPROM: 16+0-17+6 (n=3), 18+0-19+6 (n=3), 20+0-21+6 (n=6), and 22+0-22+6 weeks’ gestation livebirth status (n=4).

‡Excludes pregnancies with termination for medical reasons.

§Includes pregnancies with termination for medical reasons and missing discharge status.

In the immediate period after PPROM, the risk of birth was high: 27% (60/223) of births occurred within 72 hours of PPROM and a further 12% (27/223) by seven days after PPROM (table 4). Among those women who remained pregnant, the risk of birth was 21% (29/136) in the second week after PPROM and about 16% from the third week onwards per week. This pattern was consistent across the gestational ages at PPROM (online supplemental figure A3).

**Table 4:** Time between preterm prelabour rupture of membranes and birth in singleton pregnancies with expectant management

Time between PPROM and birth	Whole cohort (n=223)*	Gestational age at PPROM (weeks+days)†			
		16+0-17+6 (n=43)	18+0-19+6 (n=70)	20+0-21+6 (n=80)	22+0-22+6 (n=30)
<72 hours	60 (27)	16 (37)	18 (26)	20 (25)	6 (20)
72 hours-<7 days	27 (12)	4 (9)	8 (11)	9 (11)	6 (20)
7 days-<28 days	48 (22)	6 (14)	12 (17)	24 (30)	6 (20)
≥28 days	85 (38)	17 (40)	32 (46)	26 (33)	10 (33)
Not specified	3 (1)	0 (0)	0 (0)	1 (1)	2 (7)

Data are number (%). Terminations of pregnancy (n=103) were excluded from this table. PPROM=preterm prelabour rupture of membranes.

*Gestational age at PPROM 16+0-22+6 weeks+days.

†P value was 0.30 compares latency between PPROM and birth by gestational age category at PPROM, calculated with χ^2^ test.

PPROM, preterm prelabour rupture of membranes.

### Maternal outcomes

Among the 364 women within the study, five became severely unwell; two died and three were admitted to the intensive treatment unit and survived. The rate of maternal death was therefore 0.55% (2/364, 95% confidence interval 0.15% to 1.98%). Both deaths were attributed to sepsis, and the remaining three severely unwell women also had sepsis, with massive transfusion in one woman. Surgical removal of the placenta was required in four of these five women, three had cervical cerclage and two had multiple pregnancies. Three of the five women deteriorated within five days of a diagnosis of PPROM, one 1-3 weeks after PPROM and the third >3 weeks after PPROM. Further details are not reported because of the reasonable risk that the women who became seriously unwell might be identifiable, contravening the ethical approval for this anonymous population based study (deductive disclosure).

The rate of maternal sepsis was 12% (39/326) among women with singleton pregnancies and 29% (11/38) in among women with multiple pregnancies (P=0.004, table 5). In women with singleton pregnancies, the rate of sepsis was 13% (33/264) among those that initially opted for expectant management and 10% (6/62) among those who initially opted for termination of pregnancy for medical reasons (P=0.54). We could not assess the difference in rate of maternal sepsis by management choice in multiple pregnancies because only two women with multiple pregnancies opted for termination of pregnancy for medical reasons without expectant management.

**Table 5:** Maternal complications of early preterm prelabour rupture of membranes

Maternal outcomes	Sepsis		Surgery for removal of placenta	
	No (%) (95% CI)	P value	No (%) (95% CI)	P value
Whole cohort (n=364)	50 (14) (11 to 18)	—	71 (20) (16 to 24)	—
Singleton pregnancies:				
All (n=326)	39 (12) (9 to 16)	—	65 (20) (16 to 25)	—
Termination for medical reasons with no expectant management (n=62)	6 (10) (5 to 20)	0.54	8 (13) (7 to 23)	0.12
All with initial expectant management (n=264)	33 (13) (9 to 17)		57 (22) (17 to 27)	
Termination for medical reasons after expectant management (n=41)	13 (32) (20 to 47)	—	6 (15) (7 to 28)	—
Expectant management throughout pregnancy (n=223)	20 (9) (6 to 13)	—	51 (23) (18 to 29)	—
Multiple pregnancies:				
All (n=38)	11 (29) (17 to 45)	0.004*	6 (16) (7 to 30)	0.54*
Termination for medical reasons after expectant management (n=10)†	6 (60) (31 to 83)	—	3 (30) (11 to 60)	—
Expectant management throughout pregnancy (n=26)	5 (19) (8 to 36)	—	3 (12) (4 to 27)	—

Data are number (%) (95% confidence interval (CI)).

*Compared with all singleton pregnancies.

†Only two women with multiple pregnancies opted for termination of pregnancy for medical reasons without expectant management (details not shown because of small sample size).

Among the 33 women with singleton pregnancies who developed sepsis after initial expectant management, median time from PPROM to a diagnosis of sepsis was two days (interquartile range 1-8, range 0-85). The rate of maternal sepsis was higher with cervical cerclage: 34% (10/29) developed sepsis compared with 12% (40/335) without cervical cerclage (P=0.003). Surgery for removal of the placenta was performed in 20% (71/364) of pregnancies; this rate did not differ between singleton and multiple pregnancies, or between pregnancies requiring or not requiring termination for medical reasons (table 5). The rates for maternal sepsis and surgery for removal of the placenta were consistent across gestational ages (data not shown).

### Singleton baby outcomes

#### Singleton baby losses

Among 326 women with singleton pregnancies, 256 (79%) had a baby loss. A further 16 (5%) of women had a baby was live born at ≥22 weeks' gestation but their discharge status from hospital was not known. Causes of baby loss were termination of pregnancy for medical reasons (32%, 103/326), birth or intrauterine death before 22+0 weeks' gestation (ie, miscarriage, 28%, 90/326), intrauterine death at ≥22+0 weeks' gestation (11%, 35/326), and neonatal death (9%, 28/326) (table 2).

When PPROM occurred before 22+0 weeks' gestation, the rate of birth or intrauterine death (often called miscarriage) was relatively consistent (27-37%). The unadjusted relative rate ratio of intrauterine death at ≥22+0 weeks' gestation was 1.59 (95% confidence interval 1.27 to 1.99) for each additional week of gestation at PPROM, and we also saw a trend towards an increased rate of neonatal death as gestational age at PPROM increased from 16 to 22 weeks' gestation (table 2). This finding was largely because 39% of pregnancies ended within a week of PPROM (table 4), and so babies born after PPROM at 22+0 weeks' gestation had a higher risk of birth with extreme prematurity.

#### Live birth rate

Among the singleton pregnancies that had expectant management, the overall rate of live births was 44% (98/223) (table 3). If all pregnancies with termination of pregnancy for medical reasons could have been live born, the live birth rate would be 62% (201/326). In the worst case scenario with no live births in the cohort of pregnancies with termination of pregnancy for medical reasons, the live birth rate estimate would be only 30% (98/326).

Gestational age at PPROM was an important factor associated with neonatal outcomes of expectantly managed pregnancies. The live birth rate increased from 33% (14/43) in pregnancies with PPROM at 16+0-17+6 weeks' to 67% (20/30) in pregnancies with PPROM at 22+0-22+6 weeks’ gestation (table 3). This finding gives an unadjusted relative rate ratio of live birth of 1.25 (95% confidence interval 1.08 to 1.45, P=0.004) for each additional week of gestational age at PPROM. Median gestational age at birth of the 98 liveborn babies was 28+2 weeks (interquartile range 25+2-30+2).

#### Survival to hospital discharge

The overall rate of survival of babies to discharge from hospital with expectant management was 26% (54/207) when all data needed to assess this outcome were available. The range based assumption related to termination of pregnancy for medical reasons and missing data was 53% (173/326) for the best case scenario and 17% (54/326) for the worst case scenario (all terminations for medical reasons and pregnancies with missing data having an adverse outcome). We saw a trend towards improved survival with advancing gestational age at PPROM (P=0.057) (table 3). The rate of survival to discharge from hospital among liveborn babies was 55% (54/98). A further 16% (16/98) of liveborn babies had missing data for discharge status. Median gestational age at birth of the 54 babies with known survival to discharge from hospital was 29+4 weeks (interquartile range 27+1-34+2). Median length of hospital stay after birth for the 54 surviving babies was 59 days (interquartile range 17-100).

#### Morbidity among surviving neonates of singleton pregnancies

Among the 54 singleton liveborn neonates with known survival to discharge from hospital, 16 (30%) met our criteria for severe morbidity (grade 3 or 4 intraventricular haemorrhage or requiring supplemental oxygen treatment at 36 weeks' postmenstrual age, or both, table 3). The rate of survival without severe morbidity among all expectantly managed singleton pregnancies with known outcomes was 18% (38/207). The best case scenario, if all pregnancies with termination of pregnancy for medical reasons and missing data had favourable outcomes, was a survival to discharge without severe morbidity rate of 48% (157/326); the worst case scenario, if all pregnancies with termination of pregnancy for medical reasons and missing data died, was a survival to discharge without severe morbidity rate of 12% (38/326) (table 3).

Morbidity included grade 3 or 4 intraventricular haemorrhage in 11% (6/54) of surviving babies and severe lung disease after birth in 52% (28/54). Two neonates had limb contractures, affecting one and two limbs, respectively, and no surviving babies had neonatal seizures. We found no difference in morbidity according to gestational age at PPROM, but the small number of surviving babies with earlier gestational ages of PPROM limited statistical power. The rate of survival without severe morbidity seemed to be influenced by gestational age at birth; the rate for babies born before 28+0 weeks' gestation was 53% (8/15) and the rate for those born at ≥34+0 weeks' gestation was 87% (13/15) (online supplemental table A3). The unadjusted relative rate ratio of survival without severe morbidity was 1.23 (95% confidence interval 1.04 to 1.47, P=0.03) for each additional week of gestation at birth.

### Multiple pregnancy baby outcomes

Our cohort included 38 women with multiple pregnancies (10%, 38/368), which is an over-representation because <2% of births nationally are from multiple pregnancies.^10^ Twenty three were dichorionic-diamniotic twins, 10 were monochorionic-diamniotic twins, and one was trichorionic triplets. Chorionicity was not determined in four twin pregnancies.

#### Survival to discharge in multiple pregnancies

In six of 30 twin pregnancies with expectant management, both babies survived to discharge from hospital (20%). In another five twin pregnancies (17%), one baby survived to discharge from hospital (table 6). If all babies with termination of pregnancy for medical reasons had been live born, and if those with missing information for hospital discharge survived, the survival to hospital discharge rate could be as high as 27% for both babies and 46% for one twin baby at discharge. In the worst case scenario, with no live births in the cohort of pregnancies with termination for medical reasons and all those with missing discharge status having died, the survival to hospital discharge rate would be 16% for both babies, with an additional 14% of pregnancies having a liveborn single baby surviving to discharge. Most of the twin survivors were from dichorionic-diamniotic pregnancies (table 6).

**Table 6:** Perinatal outcomes for twin pregnancies

Perinatal outcomes	All twin pregnancies (n=37)†	Chorionicity	
		DCDA (n=23)	MCDA (n=10)
**Live births**			
Both babies:			
No/total No (%) expectant management‡	14/34 (41)	10/21 (48)	3/9 (33)
Worst-best possible outcome (range) (minimum No/total No (%) to maximum No/total No (%))§	14/37 (38) to 17/37 (46)	10/23 (43) to 12/23 (52)	3/10 (30) to 4/10 (40)
Single baby:			
No/total No (%) expectant management‡	3/34 (9)	1/21 (5)	2/9 (22)
Worst-best possible outcome (range) (minimum No/total No (%) to maximum No/total No (%))§	3/37 (8) to 12/37 (32)	1/23 (4) to 6/23 (35)	2/10 (20) to 4/10 (40)
**Survival to hospital discharge**			
Both babies:			
No/total No (%) expectant management‡*	6/30 (20)	5/19 (26)	1/8 (13)
Worst-best possible outcome (range) (minimum No/total No (%) to maximum No/total No (%))§	6/37 (16) to 10/37 (27)	5/23 (22) to 7/23 (35)	1/10 (10) to 2/10 (20)
Single baby:			
No/total No (%) expectant management‡*	5/30 (17)	3/19 (16)	2/8 (25)
Worst-best possible outcome (range) (minimum No/total No (%) to maximum No/total No (%))§	5/37 (14) to 17/37 (46)	3/23 (13) to 10/23 (43)	2/10 (20) to 5/10 (50)
**Termination for medical reasons**			
No/total No (%) terminations for medical reasons of both fetuses	3/37 (8)	2/23 (9)	1/10 (10)
No/total No (%) terminations for medical reasons of one fetus	6/37 (16)	5/23 (22)	1/10 (10)

DCDA=dichorionic-diamniotic; IQR=interquartile range; MCDA=monochorionic-diamniotic.

*Excludes pregnancies with babies that were live born at ≥22+0 weeks' gestational age with missing discharge status: dichorionic-diamniotic discharge status missing for both babies (n=2); monochorionic-diamniotic discharge status missing for one baby (n=1); unknown chorionicity discharge status missing for one baby (n=1).

†Four twin pregnancies with unreported chorionicity were included in the all twin pregnancies data only. The one triplet pregnancy was excluded from this table.

‡Excludes pregnancies with termination for medical reasons.

§Includes pregnancies with termination for medical reasons and missing discharge status.

Among the 10 monochorionic-diamniotic pregnancies, only one pregnancy resulted in survival of both babies to discharge, and another two pregnancies had survival of one baby. Six of the 10 monochorionic-diamniotic pregnancies had either laser coagulation or amnioreduction for twin-to-twin transfusion syndrome before PPROM.

#### Survival to discharge without severe morbidity in multiple pregnancies

Among the 17 (17/54, 31%) of 54 babies who survived to discharge from hospital after expectant management of a twin pregnancy, four babies (4/17, 24%) had severe morbidity when discharged. These were all from dichorionic-diamniotic pregnancies. The rate of survival to discharge from hospital without severe morbidity after expectant management of twin pregnancies was therefore 13/54 (24%) babies (four sibling pairs and five single twins).

## Discussion

### Statement of principal findings

The results of this national, population based study of PPROM before 23 weeks' gestation highlight the diverse perinatal and maternal outcomes of this condition. Maternal sepsis developed in 14% (50/364) of women and two died. Although 26% (54/207) of women with expectantly managed singleton pregnancies had a baby that survived to discharge from hospital, only 18% (38/207) of babies survived without severe morbidity. Most neonatal morbidity and mortality were related to extreme prematurity, secondary to 39% of births within a week of PPROM. Uncertainty exists for perinatal outcomes because 32% of pregnancies were terminated for medical reasons.

### Comparison with previous studies

The two maternal deaths gave a rate of 549 per 100 000 maternities with PPROM at 16+0-22+6 weeks’ gestation (95% confidence interval 151 to 1981 per 100 000). This rate is considerably higher than the baseline UK maternal mortality rate of 11 per 100 000 maternities,^15^ and comparable with the risk of death after admission to hospital with SARS-CoV-2 infection when pregnant (406 per 100 000)^16^ or having a pulmonary embolism in pregnancy (3413 per 100 000).^17^

In the past decade, more than 700 women have been included in observational cohorts of PPROM at similar gestational ages with no maternal deaths reported.^3 18–24^ This finding concurs with no maternal deaths reported in the most recent review on this topic,^25^ and only one maternal death reported in the largest review, published in 2009, citing a publication from 1988.^2 26^ The key difference from previous studies is that our cohort was population based because we surveyed all 194 consultant led maternity units in the UK in contrast with previously published studies from five or fewer (often specialised) centres. In accordance with our population based approach, second trimester PPROM associated with maternal sepsis and death has featured in three of the UK's maternal mortality reports in the past decade.^14 26 27^ Population level data have also identified seven maternal deaths after PPROM at 14+0-24+6 weeks' gestation from 2001 to 2015 in France, giving an estimated risk of death of 45 per 100 000 maternities with this complication.^27^ Similar causes of death to our cohort were found: six were attributed to sepsis and one to haemorrhage secondary to placenta accreta spectrum. Hence the population based literature suggests that these deaths are not isolated incidents and might have previously been under-reported, potentially because the literature has been based on data from a small number of centres. Also, studies might have tended to preferentially report on cohorts with favourable outcomes, with possible positive publication bias, which is much less likely in population based studies.

The rate of maternal infectious morbidity in previous studies of expectantly managed singleton pregnancies was similar to our value of 12%.^3 14 18^ In our cohort, maternal sepsis was higher in twin pregnancies, also in agreement with previous work.^28^ Similar to current clinical expertise and one randomised controlled trial that was ended early, maternal sepsis was higher with cervical cerclage.^29 30^ Surgery for removal of the placenta is a less well recognised complication of birth after early PPROM but occurred in 20% of our cohort, including four of the five women who became severely unwell. This rate concurs with a cohort with PPROM in mid-trimester in Ireland.^3^ The combination of requiring surgery for removal of the placenta and sepsis should alert clinicians to the possibility of maternal deterioration.

Even if all women in our study had opted for termination of pregnancy for medical reasons without expectant management, and if termination for medical reasons had avoided all incidences of maternal sepsis after termination was performed, a reasonable expectation is that more than half of the instances of maternal sepsis would still have developed. In our cohort, 10% of women who opted for immediate termination of pregnancy for medical reasons with singleton pregnancies developed sepsis. This finding agrees with a retrospective case control study in the US of 174 pregnances with PPROM at 14+0-22+6 weeks' where expectant management was not associated with an increase in composite morbidity (including sepsis and endometritis) compared with immediate termination of pregnancy for medical reasons.^31^ We suggest that this finding is because sepsis can develop quickly. Infection could even have contributed to the occurrence of PPROM whereas termination of pregnancy for medical reasons takes time to arrange and perform. Among the 33 women with singleton pregnancies that developed sepsis after initial expectant management, median time from PPROM to a diagnosis of sepsis was two days (interquartile range 1-8) and among the 62 singleton pregnancies that were terminated for medical reasons without expectant management, median time from PPROM to termination of pregnancy for medical reasons was also two days (1-3.5).

The perinatal survival to hospital discharge rate of 26% for expectantly managed singleton pregnancies, and the range of possible survival (17-53%) when pregnancies with termination of pregnancy for medical reasons and unknown outcomes were accounted for, were similar to observational studies over the past decade that reported rates of 17-40% with similar gestational ages of PPROM.^18 20 24 32–36^ Among the babies that survived to discharge from hospital, 70% (38/54) avoided severe morbidity by our definition, in keeping with recently published observational studies.^14 18 24 34 35^

The definition of severe neonatal morbidity was chosen for consistency with Kibel et al^14^ who related neonatal outcomes at hospital discharge after pregnancies complicated by PPROM at 20-24 weeks' gestation to outcomes at a corrected age of 18-21 months. Of 24 babies with grade 3 or 4 intraventricular haemorrhage, requiring oxygen at 36 weeks' postmenstrual age, or with ≥grade 3 retinopathy of prematurity, or a combination of these conditions, eight (30%) had moderate-to-severe morbidity at age 18-21 months. Among 27 babies who were born after pregnancies complicated by PPROM at 20-24 weeks' gestation but who did not have any intraventricular haemorrhage, retinopathy of prematurity, or require oxygen at 36 weeks' postmenstrual age, none had moderate-to-severe morbidity at age 18-21 months.^14^ Our results could indicate long term moderate-to-severe morbidity in a similar proportion of children, although we did not collect data about retinopathy of prematurity so the burden of severe morbidity might be slightly underestimated.

Women who had expectant management after PPROM at 16+0-17+6 weeks’ gestation and gave birth after 23 weeks' gestation had comparable live births and perinatal morbidity rates with those who had PPROM at 22+0-22+6 weeks’ gestation. This finding could be a result of lack of statistical power because only seven babies survived to discharge after PPROM at 16+0- 17+6 weeks' gestation. Also, when PPROM occurred earlier in pregnancy, possibly more pregnancies with less favourable characteristics had a termination of pregnancy for medical reasons, negating any negative effect of gestational age of PPROM on perinatal morbidity. A study from the Netherlands, however, with a rate of termination of only 2%, also found no difference in perinatal morbidity after PPROM at ≥13-<20 weeks' gestation (n=21) compared with ≥20-<24 weeks' gestation (n=41).^34^ We suggest that the effect of gestation on perinatal morbidity after PPROM needs further evaluation.

Median gestational age at birth of babies surviving to discharge from hospital was 29+4 weeks, and median length of hospital stay after birth for surviving babies was 59 days (interquartile range 17-100). This length of stay in hospital is shorter than in recent studies from Australia^20^ (median 76 days, interquartile range 44-111) and Japan^37^ (mean 155 days, standard deviation 53), but is still a substantial amount of time and likely to have a major effect on the whole family in the medium term. We suggest this information should be included in patient counselling.

As gestational age at birth advanced, the possibility of live birth and perinatal survival to discharge from hospital improved, as expected.^2^ Among those born after 34 weeks' gestation, however, two babies (2/15, 13%) had severe morbidity, highlighting the complexities of these cases and the need for ongoing multidisciplinary team care, including neonatologists, even at relatively advanced gestations.

Babies of dichorionic-diamniotic multiple pregnancies with PPROM seemed to have comparable pregnancy outcomes with singletons, similar to previous studies.^38^ Monochorionic-diamniotic twin pregnancies had lower perinatal survival, but 60% of these pregnancies also had pathologies of monochorionicity, such as twin-to-twin transfusion syndrome and selective growth restriction. Therefore, the pathologies unique to monochorionic pregnancies are likely to be important contributors to mortality in these instances.

### Strengths and limitations

The UKOSS infrastructure allowed us to conduct a large population based study of PPROM before 23 weeks' gestation. Unavoidable uncertainty exists, however, for perinatal outcomes because 31% (113/364) of the population opted for termination of pregnancy for medical reasons and we could not follow-up 16 liveborn babies (5% of all singleton births), particularly those that moved to another hospital as part of their care. The UKOSS infrastructure does not facilitate long term follow-up of babies into childhood, and therefore the information most wanted by parents, on the longer term outcomes of offspring, is not available with this methodology. Also, we defined neonatal morbidity as intraventricular haemorrhage grade 3 or 4, or requiring supplemental oxygen at 36 weeks' postmenstrual age, or both, which does not rule out other morbidities.

The incidence of PPROM between 16+0 and 22+6 weeks’ gestation of 0.04% was likely underestimated because of under-reporting, particularly during the covid-19 pandemic. We have no evidence of biased reporting, however, which might influence the generalisability of the results. A recent analysis of hospital episode statistics for the whole of France found a prevalence of 0.2% of PPROM at 14+0-24+6 weeks' gestation, which was considered to be likely underestimated because to fully validate the diagnosis with coded data is not possible.^27^ Within our study the incidence of PPROM was lowest between July and December 2020, possibly because of staffing pressures or potentially a lower rate of PPROM secondary to public health control measures, such as lockdowns.

The UKOSS methodology requires reporting clinicians to identify eligible cases. This approach might favour identification of pregnancies with a longer time between PPROM and birth, likely requiring a longer stay in hospital, which in turn could bias the results towards pregnancies with a more favourable neonatal outcome. In our study, median time between PPROM and birth for expectantly managed singleton pregnancies was 13 days (interquartile range 3-50, n=220). This value is in the higher range of other studies focusing on PPROM before 24 weeks' gestation that reported times of seven days (interquartile range 3-29, n=74),^18^ 11.5 days (3-29, n=130),^20^ 13 days (1-85, n=37),^3^ and 14 days (3-32, n=46).^22^ Nevertheless, in 27% of expectantly managed pregnancies, babies were born in the 72 hours after PPROM, indicating that the methodology also identified shorter times between PPROM and birth.

This study was prompted by the lack of national or international guidelines to standardise care after PPROM before 23 weeks' gestation. Women in this study likely received a variety of models of care based on their own and their clinician's preference (eg, provision of antibiotics, monitoring, and steroids). These practices could have influenced pregnancy outcomes, and analysis of these data will form a separate paper. We have presented general outcome categories to give clinicians, patients, and their families dealing with PPROM before 23 weeks' gestation a broad framework to guide counselling.

To simplify case reporting and capture the maximum number of incidences of PPROM, the inclusion criteria were brief, and questions about fetal anomalies, whether identified at the time of PPROM or not, were not included. The survey reporters were not asked whether women who opted for a termination of pregnancy for medical reasons, or had a baby loss, also had a fetus with a life threatening anomaly. Fetal anomalies might have been present in some instances, which could have influenced the decision to terminate the pregnancy. We collected a limited range of neonatal outcomes, and no outcomes after hospital discharge because of resource constraints.

### Implications for clinical practice

In this study, we showed that pregnancies with PPROM before 23 weeks' gestation had a high risk of perinatal and maternal morbidities. Individual obstetricians are likely to see only one or two instances of early PPROM a year, and therefore cannot gain substantial experience relevant to the condition. Similar to other studies, we found that not all women opting for termination of pregnancy for medical reasons avoided severe morbidity.^28 39^

In current UK practice, pregnancies before 20 weeks' gestation are often cared for on gynaecology rather than obstetric wards. In our study, perinatal mortality was largely attributable to birth before viability and extreme prematurity because 39% of births occurred in the week after PPROM, and a further 22% in the following week. This pattern was consistent across the gestational ages of PPROM, and was similar to previous studies.^18 34^ We suggest that pregnancies with PPROM before 23 weeks' gestation are likely to need the support of neonatal teams, maternal critical care, and bereavement teams. Obstetricians with an interest in this condition, together with dedicated midwives, are possibly best placed to coordinate this care immediately after PPROM and for the remainder of the pregnancy. About half of the babies who were admitted to neonatal units after PPROM between 16+0 and 22+6 weeks’ survived. Most survivors did not have the morbidities we reported. Among survivors, the likelihood of the morbidities we identified was lower with each week of completed pregnancy. We suggest that teams with expertise in the management of very early PPROM, along with patient representatives, work together to develop guidelines for care.

### Implications for research

This study provides baseline data on perinatal and maternal outcomes of pregnancies with PPROM before 23 weeks' gestation. Studies attempting to improve outcomes can build on our data. Interventions could be new treatments aimed at treating the pathology, or care bundles, including training, to optimise care and the timing of delivery with interventions already available. Materials for families with very early PPROM need to consider the inherent uncertainty within the data secondary to 32% of women with singleton pregnancies opting for termination of pregnancy for medical reasons. The optimal ways to communicate these uncertainties within the data to a wider audience, and support families with such complex pregnancies, have yet to be determined.

We can only comment on severe morbidity in babies at discharge from hospital, and previous work suggests that 70% of these babies will not have major morbidity when aged 18-21 months.^14^ The information most wanted by prospective parents is the rate of long term disability in offspring. Future research needs to incorporate ways of obtaining this information. Substantial unmeasured psychological morbidity related to this condition is likely. The optimal way of managing very early PPROM to support the psychological wellbeing of families requires further consideration.

### Conclusions

Pregnancies with PPROM before 23 weeks' gestation can have favourable maternal and pregnancy outcomes, but a substantial proportion of these pregnancies are complicated by maternal morbidity and perinatal mortality and morbidity. All clinicians who care for these families need to be aware of the risk of maternal sepsis and death. These data will be helpful in counselling families with PPROM before 23 weeks' gestation and should be incorporated into updates of clinical guidelines.

## Data Availability

No data are available. Data cannot be shared publicly because of confidentiality issues and potentially identifiable sensitive data as identified within the Research Ethics Committee application/approval. Requests to access the data can be made by contacting the National Perinatal Epidemiology Unit data access committee via general@npeu.ox.ac.uk
