# Supplementary material for: Preterm prelabour rupture of membranes before 23 weeks’ gestation: prospective observational study
Source: BMJ Med. 2024 Mar 19;3(1):e000729. doi: 10.1136/bmjmed-2023-000729 (PMC11005708; doi:10.1136/bmjmed-2023-000729)
Supplement: Supplementary data [file bmjmed-2023-000729supp001.pdf]

Appendix 1 for UKOSS PPROM

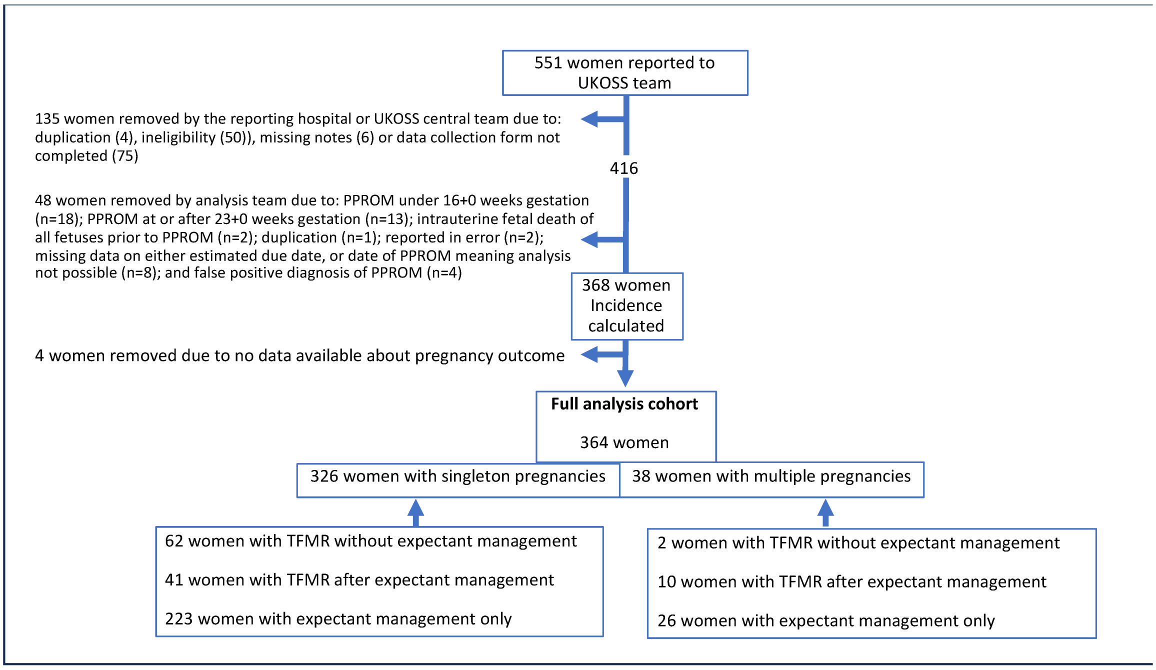

Figure A1: Participant flow through study. TFMR, Termination For Medical Reasons. UKOSS, UK Obstetric Surveillance System. False positive diagnosis of PPROM was defined as women in which all follow-up ultrasound scans showed normal liquor volume and birth was at or after 37+0 weeks' gestational age

COVID-19 Pandemic

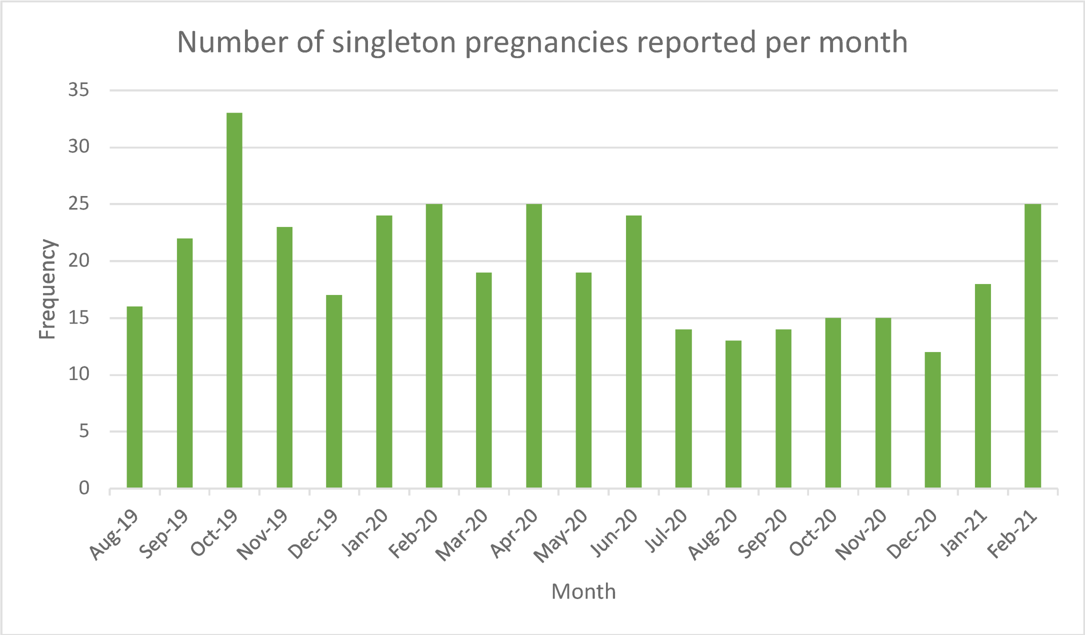

Figure A2: Number of singleton pregnancies reported to the study by month

Maternal outcomes of all singleton pregnancies	Prior to COVID-19	During COVID-19	P value
Number	137	189	
Sepsis	15 (11%)	24 (13%)	0.244
Surgery for placental removal	26 (19%)	39 (21%)	0.712
ITU admission	0 (0%)	2 (1%)	0.230
Death	0 (0%)	2 (1%)	0.230

Table A1: Maternal outcomes of singleton pregnancies according to whether preterm premature rupture of membranes occurred prior to or during the COVID-19 pandemic (which commenced in March 2020).

Infant outcomes of all singleton pregnancies	Prior to COVID-19 pandemic	During COVID-19 pandemic	P value
Number	137	189	
Termination of pregnancy	45 (33%)	58 (31%)	0.434
Pregnancy loss, stillbirth, or livebirth with neonatal death (n, %)	65 (47%)	88 (47%)	
Livebirth with survival to hospital discharge (n, %)	23 (17%)	31 (16%)	
Livebirth with unknown discharge status (n, %)	4 (3%)	12 (6%)	

Table A2: Infant outcomes of singleton pregnancies according to whether preterm premature rupture of membranes occurred prior to or during the COVID-19 pandemic (which commenced in March 2020).

Pregnancy after PPROM

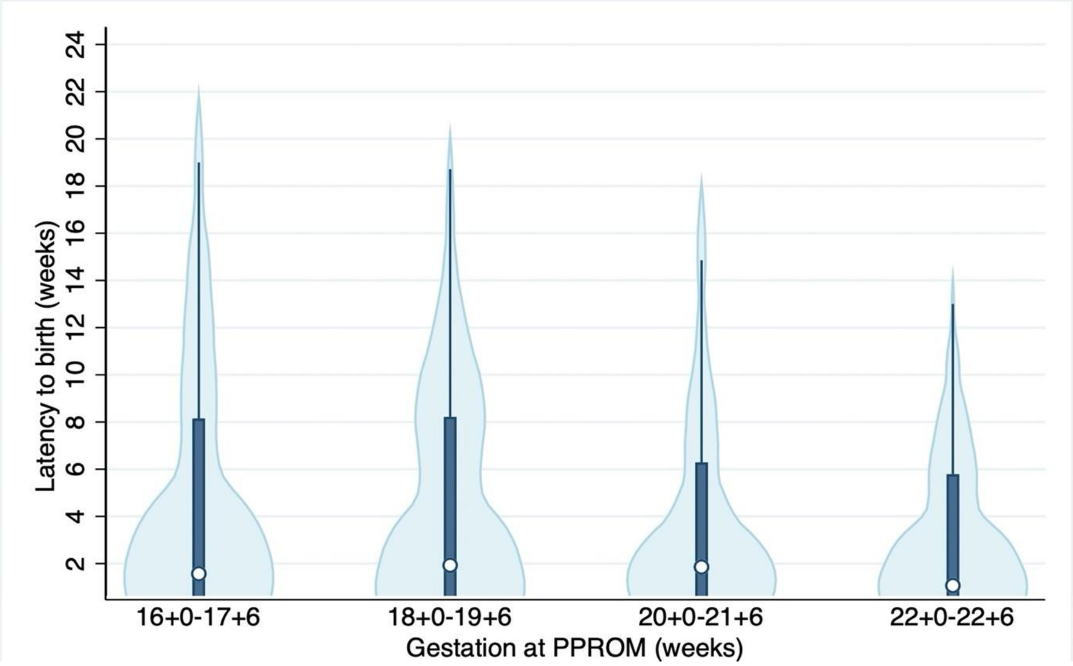

Figure A3: Violin plot illustrating the latency between PPROM and birth for women with conservative management and singleton pregnancies (n=223). The white dot shows the median value (1 week 6 days for the whole group) the dark bar shows the IQR (3 days to 7 weeks 1 day for the whole group). The thin blue line shows 1.5x the interquartile range. The light blue area represents the kernel density estimation to show the distribution shape of the data. The plot has been truncated at 0 weeks.

Severe morbidity by gestational age at birth

Percentatge of surviving infants that did not have severe morbidity n=54	**Gestation at birth**											
	**Under 24 weeks**		**24+0-27+6 weeks**		**28+0-31+6 weeks**		**32+0-33+6 weeks**		**34+0-36+6 weeks**		**37+0 or over**	
Survival without severe morbidity	1/4	25%	7/11	64%	15/22	68%	2/2	100%	10/12	83%	3/3	100%
	(0	-67%)	(35	-92%)	(49	-88%)			(62	-100%)		

Table A3: Number of surviving infants that did not have severe morbidity by gestational age at birth in singleton pregnancies. Presented as n/N, % and 95% confidence interval
